# Volatile allosteric antagonists of mosquito odorant receptors inhibit human-host attraction

**DOI:** 10.1074/jbc.RA120.016557

**Published:** 2020-12-18

**Authors:** Georgia Kythreoti, Nadia Sdralia, Panagiota Tsitoura, Dimitrios P. Papachristos, Antonios Michaelakis, Vasileios Karras, David M. Ruel, Esther Yakir, Jonathan D. Bohbot, Stefan Schulz, Kostas Iatrou

**Affiliations:** 1 Institute of Biosciences and Applications, National Centre for Scientific Research “Demokritos”, Aghia Paraskevi, Greece; 2 Entomology and Agricultural Zoology, Benaki Phytopathological Institute, Kifissia, Greece; 3 Department of Entomology, The Robert H. Smith Faculty of Agriculture, Food and Environment, The Hebrew University of Jerusalem, Rehovot, Israel; 4 Institute of Organic Chemistry, Technische Universität Braunschweig, Braunschweig, Germany

**Keywords:** allosteric regulation, cell surface receptor, 7-helix ligand-gated channel, ion channel, ligand-binding protein, mosquito odorant receptors, ORco coreceptor, volatile organic compounds, evolutionary conservation, mosquito repellents, ligand-binding sites, Aaeg and *Ae. aegypti*, *Aedes aegypti*, Aalb and *Ae. albopictus*, *Aedes albopictus*, Agam and *An. gambiae*, *Anopheles gambiae*, CA, cumin alcohol, CAR, (1*S*)-3-carene, CRV, carvacrol, DCM, dichloromethane, DEET, *N,N*-diethyl-3-methylbenzamide, IPC, isopropyl cinnamate, LA, linalyl acetate, OA, ORco agonist, OBP, odorant-binding protein, OCT, (2*E*,4*E*)-2,4-octadienal, OR, odorant receptor, ORco, odorant receptor common subunit, ORx, variable ligand-binding odorant receptor subunit, VOC, volatile organic compound

## Abstract

Odorant-dependent behaviors in insects are triggered by the binding of odorant ligands to the variable subunits of heteromeric olfactory receptors. Previous studies have shown, however, that specific odor binding to ORco, the common subunit of odorant receptor heteromers, may allosterically alter olfactory receptor function and profoundly affect subsequent behavioral responses. Using an insect cell–based screening platform, we identified and characterized several antagonists of the odorant receptor coreceptor of the African malaria vector *Anopheles gambiae* (AgamORco) in a small collection of natural volatile organic compounds. Because some of the identified antagonists were previously shown to strongly repel *Anopheles* and *Culex* mosquitoes, we examined the bioactivities of the identified antagonists against *Aedes*, the third major genus of the Culicidae family. The tested antagonists inhibited the function of *Ae. aegypti* ORco *ex vivo* and repelled adult Asian tiger mosquitoes (*Ae. albopictus*). Binary mixtures of specific antagonists elicited higher repellency than single antagonists, and binding competition assays suggested that this enhanced repellence is due to antagonist interaction with distinct ORco sites. Our results also suggest that the enhanced mosquito repellency by antagonist mixtures is due to additive rather than synergistic effects of the specific antagonist combinations on ORco function. Taken together, these findings provide novel insights concerning the molecular aspects of odorant receptor function. Moreover, our results demonstrate that a simple screening assay may be used for the identification of allosteric modifiers of olfactory-driven behaviors capable of providing enhanced personal protection against multiple mosquito-borne infectious diseases.

Insect odorant receptors (ORs) are heteromeric ligand-gated ion channels expressed by olfactory receptor neurons inside olfactory sensilla (1, 2, 3). Together with odorant-binding proteins (OBPs) (4) and odorant-degrading enzymes (5) that are produced by accessory cells in the olfactory sensilla (6, 7, 8) and secreted in the lymph surrounding the olfactory receptor neurons, ORs constitute the molecular gateway to the olfactory pathway and associated behaviors that are important for survival and reproduction (9). The odorant receptor heteromeric complexes consist of an obligatory and highly conserved subunit, ORco (10, 11, 12, 13, 14), and one of many variable ligand-binding ORx subunits (12, 15) in as yet undetermined molar ratios. Odorant ligands act either as receptor agonists or antagonists in an ORx-specific manner (16, 17, 18, 19, 20, 21, 22). In cell cultures, homomeric ORco channels are formed that are activated by specific ORco agonists (OAs) such as VUAA1 and OrcoRAM2 (23, 24, 25).

In previous studies, we have utilized multiple OBPs of the African malaria mosquito vector *Anopheles gambiae* (AgamOBPs; (26, 27, 28, 29, 30, 31)) as screening tools for the discovery of natural volatile organic compounds (VOCs) capable of modifying olfaction-mediated behaviors (32, 33). This effort resulted in the identification of natural compounds with strong repellent activities against both *Anopheles* and *Culex* mosquitoes (33) suggesting the existence of phylogenetically conserved mechanisms and behavioral outputs in mosquitoes. Further studies revealed that the most potent of the identified repellents acted as allosteric inhibitors of multiple AgamOR heteromeric complexes and blocked odorant-specific responses by interacting directly with AgamORco (34). In addition, we have shown that *An. gambiae* ORx/Orco functional responses elicited by ORx-specific odor agonists were enhanced both in terms of potency and efficacy by one to two orders of magnitude in the presence of an OA (35). These findings suggested induction of conformational rearrangements in ORx ligand-bound ORx/ORco receptor complexes caused by the binding of the OA and resulting in enhanced inward currents into the receptor-expressing cells.

In view of these results and given the previously demonstrated importance of ORco for the functionality of OR heteromers and OR-dependent behaviors (36, 37, 38, 39, 40, 41, 42, 43), we have employed the lepidopteran insect cell–based assay toward the rapid detection of potential agonists and antagonists of AgamORco. This system relies on the stable expression of homomeric AgamORco in cells constitutively expressing a luminescence-emitting calcium biosensor reporter protein. Here, we report on the screening of a small collection of VOCs of plant, arthropod, and bacterial origins for the identification of modulators of AgamORco function. The screening resulted in the identification of several AgamORco-specific antagonists. Considering the high degree of phylogenetic conservation of ORco and its functional relevance, which was demonstrated by our previous findings that natural compounds inhibiting AgamORco activity were capable of repelling at least two mosquito genera, *Anopheles* and *Culex*, we examined whether the identified ORco antagonists were also active against the third major genus of Culicidae mosquitoes, *Aedes*. Two tested antagonists elicited significant inhibition of inward currents mediated by VUAA1 in *Xenopus laevis* oocytes expressing *Aedes aegypti* ORco (AaegORco). Examination of the bioactivity of the identified antagonists, as well as binary and ternary mixtures thereof, against available laboratory populations of *Aedes albopictus* elicited an avoidance behavior. Some of the mixtures caused anosmia-like effects similar to equivalent doses of N,N-diethyl-3-methylbenzamide (DEET). Antagonist binding competition assays against an OA point to the simultaneous binding of one antagonist to the OA-binding site on ORco and to one or more alternative binding sites of the other as a plausible cause for the observed enhanced activities of the binary mixtures.

## Results

**Figure 1:**
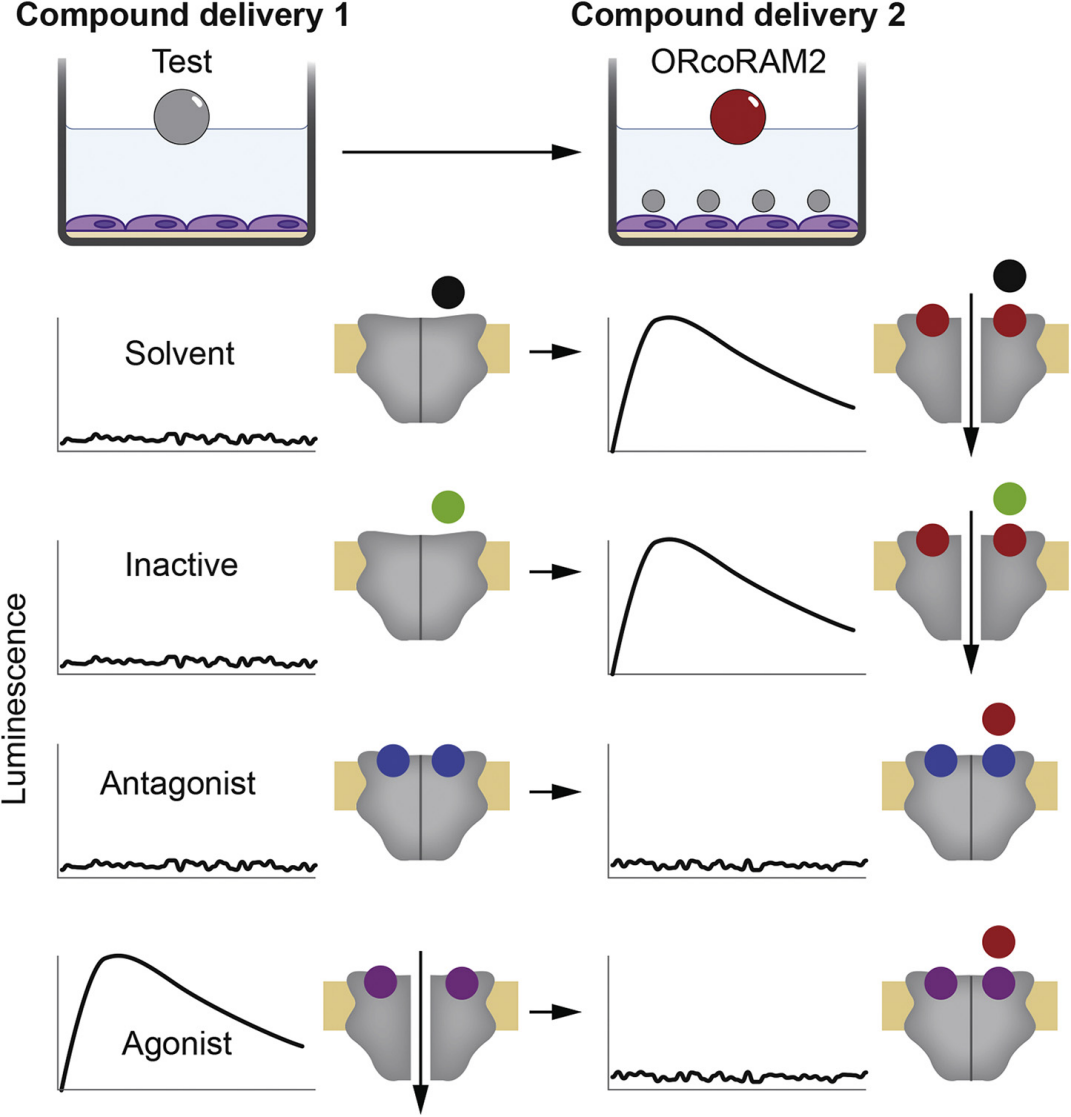
**Insect cell-based screening assay for ligand identification.** Schematic representation of a two-step screening assay for volatile organic compound (VOC) activity determination performed in a 96-well format containing lepidopteran insect cells expressing *An. gambiae* ORco functional homomeric channel and Photina Ca^2+^ biosensor. Initially, a tested VOC is added at a concentration of 100 μM and the response of the ORco channel is monitored. This is followed by addition of 100 μM ORcoRAM2, a known ORco agonist, and measurement of the secondary response. The anticipated outcomes and corresponding VOC classifications are indicated. For simplification reasons, the recently deduced homotetrameric structure of the ORco channel is illustrated here as a homodimer. Note also that, although the orthosteric binding of antagonists and new agonists in the postulated ORco agonist (VUAA1 or OrcoRAM2) site is shown in the figure, their binding in alternative, allosteric binding sites is also possible but not illustrated here. *Colored circles* define compound properties as follows: *gray*, test compound; *red*, ORcoRAM2; *black*, solvent; *green*, inactive ligand; *blue*, antagonist; *magenta*, agonist.

The screening platform employed in this study exploits the property of AgamORco homomers to form functional ligand-gated cation channels in cultured lepidopteran cells (34, 35). The constitutively expressed reporter photoprotein Photina detects the entry of Ca^2+^ ions into the cell upon AgamORco channel activation. The screening protocol, performed in a 96-well format, involved the sequential addition of a tested compound and *N*-(4-ethylphenyl)-2-{[4-ethyl-5-(3-pyridinyl)-4H-1,2,4-triazol-3-yl]thio}acetamide, the Orco Receptor Activator Molecule, ORcoRAM2, as OA, both at 100 μM concentrations, to the transformed cells (Fig. 1).

Similarly to the working scheme presented earlier (34, 35), the general principle for agonist/antagonist screening has been that the addition of ethanol solvent or a compound devoid of ORco-binding activity would allow Ca^2+^ influx resulting in cellular luminescence upon sequential addition of the ORco agonist, whereas the addition of a compound acting as an ORco antagonist would prevent, partially or completely, luminescence emission upon secondary addition of the ORco agonist. The same expression platform also allows the identification of compounds acting as ORco agonists. In that case, addition of an active compound would be expected to cause calcium ion influx and hence cell luminescence emission, whereas no response would be expected upon addition of the known agonist after the dissipation of the first luminescence burst, owing to temporary inactivation of the ORco channel (34).

**Figure 2:**
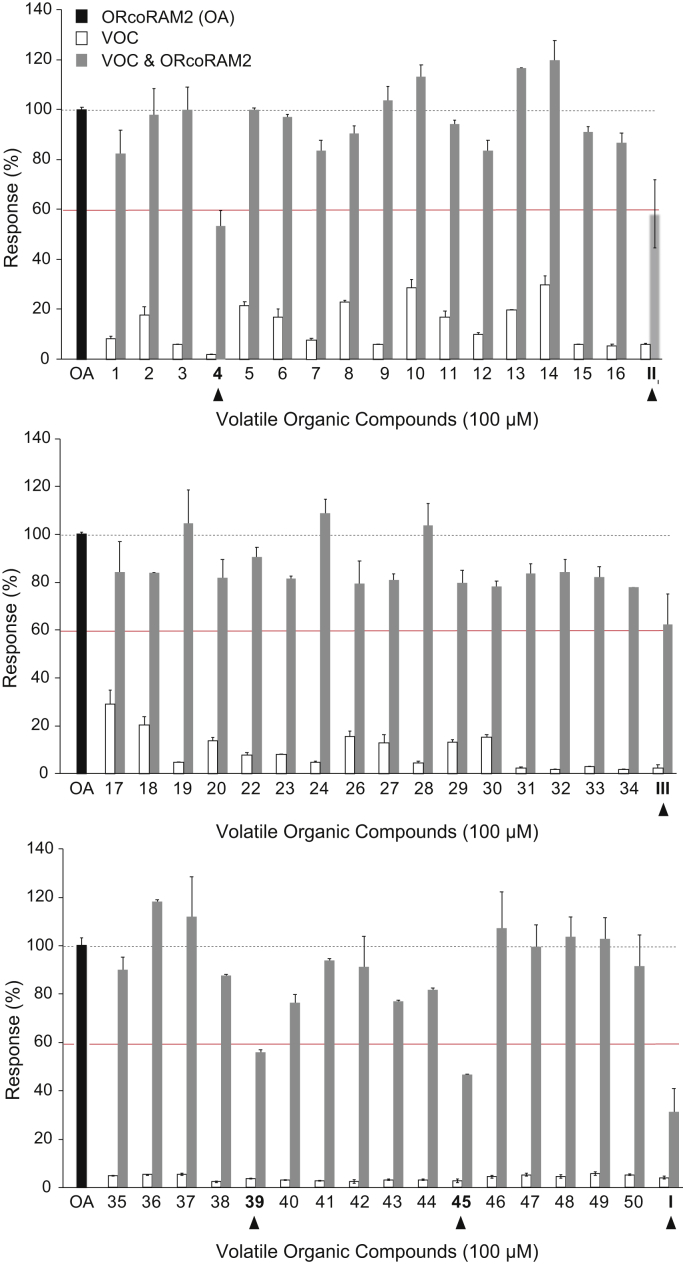
**Initial screening results.** All compounds were tested at a final concentration of 100 μM. The primary compound additions (*white bars*) do not induce any significant ORco channel function, whereas secondary additions of the OA (ORcoRAM2) to wells containing previously added, functionally inactive compounds produce responses (*gray bars*) equal to at least 80% of the full response obtained in the control wells (ORco agonist [OA] only added, shown with a *black bar* at the left of each panel). Orco antagonist hits produce significantly lower secondary responses, arbitrarily set at ≥60% of the normal channel response, upon OA addition. For putative OAs, the window of functional response to the primary addition was again arbitrarily set at ≥60% of the value of the known OA response (ORcoRAM2). Numbers correspond to those of the compounds shown in Table S1. Error bars indicate mean ± SE.

For the identification of ORco antagonist hits, the *secondary responses* to OA addition were set arbitrarily at a maximum of 60% of the normal channel response to OA addition, *i.e.*, a 40% or higher inhibition of ORco functional response. For the case of ORco agonists, the *primary responses* to the addition of the screened compounds were also set arbitrarily at 60% or greater relative to the normal channel response obtained upon addition of the known OA, OrcoRAM2 (Fig. 2).

### Natural VOCs inhibit AgamORco homomeric channel activity

The examination of 50 natural VOCs (Table S1) for the presence of AgamORco function modulators employed as control the mosquito repellent isopropyl cinnamate (IPC) (compound II; (44)) that was previously shown to act as an AgamORco channel antagonist (34). The initial screen resulted in the identification of five hits with AgamORco antagonistic activity (Fig. 2).

Two of the identified hits, carvacrol (CRV) and cumin alcohol (CA) (compounds I and III, respectively), were previously shown to be effective repellents for *An. gambiae* and *Culex* spp mosquitoes (33) and also to inhibit AgamORco activity to an extent that was not determined at the time (34). In contrast, no relevant information existed concerning the bioactivity of the other three antagonist hits, compounds #4 (linalyl acetate [LA]), #39 ((2E,4E)-2,4-octadienal [OCT]), and #45 ((1S)-3-carene [CAR]) (Fig. 2 and Table S1). No agonists inducing significant AgamORco activity were found in this VOC collection. Although several compounds such as #5, 8, 10, 13, 14, 17, and 18 (Fig. 2 and Table S1) produced, upon primary addition, notable responses suggestive of an agonist-like behavior, the observed responses ranged between 20% and 30% of that obtained upon addition of the known OA, *i.e.*, considerably lower than the 60% minimum response limit that was set for potential agonists. Although noted, these compounds have yet to be characterized further.

**Figure 3:**
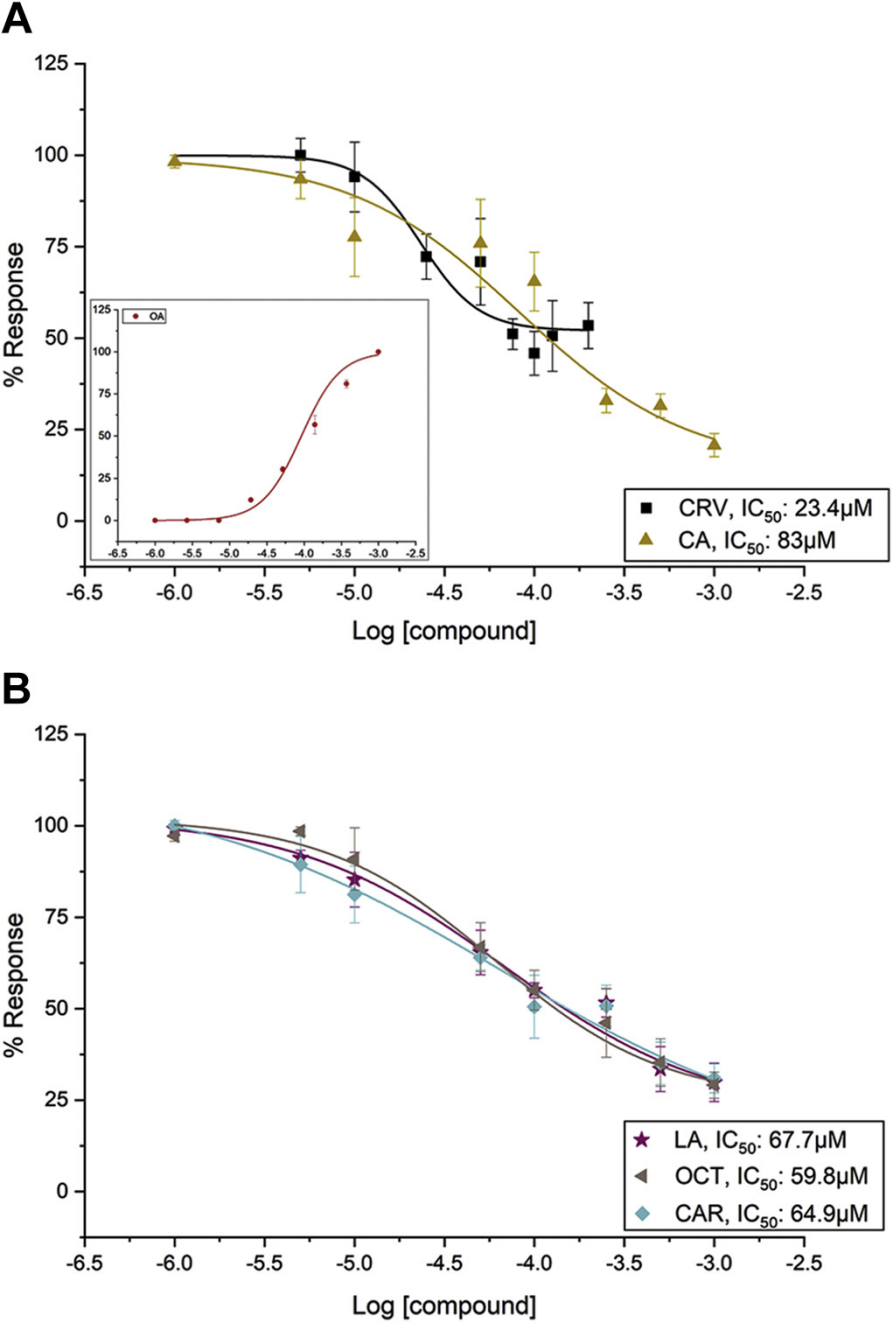
**Dose-dependent inhibition of AgamORco function by identified antagonists.** IC_50_ values for all tested compounds except carvacrol (CRV) were determined using antagonist concentrations in the range of 1 μM to 1 mM. Because CRV was found to be toxic to the cells at concentrations above 250 μM, concentrations ranging from 1 to 200 μM were used for its dose–response evaluation. *A*, the IC_50_ values determined for the two known repellents, CRV and cumin alcohol (CA), were 23.4 μM (pIC_50_: 4.63179 ± 0.09524, R^2^: 0.9755) and 83 μM (pIC_50_: 4.08243 ± 0.28481, R^2^: 0.99989), respectively, whereas that for the previously characterized ORco antagonist isopropyl cinnamate (IPC) was 41.7 μM (pIC_50_: 4.37919 ± 0.061, R^2^: 0.9883; (34)). The EC_50_ for the ORco agonist (OA) ORcoRAM2 from the curve that is shown in the *inset* is 91.9 μM (pEC_50_: 4.03684 ± 0.11567, R^2^: 0.99998). *B*, the IC_50_ values for the three new putative ORco antagonists, linalyl acetate (LA), (2*E*,4*E*)-2,4-octadienal (OCT), and (1S)-3-carene (CAR), were 67.7 μM (pIC_50_: 4.16927 ± 0.15954, R^2^: 0.99999), 59.8 μM (pIC_50_: 4.22309, R^2^: 0.99988), and 64.9 μM (pIC_50_: 4.18725 ± 0.31571, R^2^: 0.99998), respectively. Error bars indicate mean ± SE. Data points were normalized to the maximum value and multiplied by 100.

A quantitative assessment of the effects of the antagonist hits on AgamORco channel function was undertaken by determining the inhibition in the OA-dependent channel activity by increasing antagonist concentrations. As is depicted by the dose–response curves presented in Figure 3, all five hits were found to antagonize AgamORco channel function in a dose-dependent manner with IC_50_ values ranging from 23 to 83 μM.

**Figure 4:**
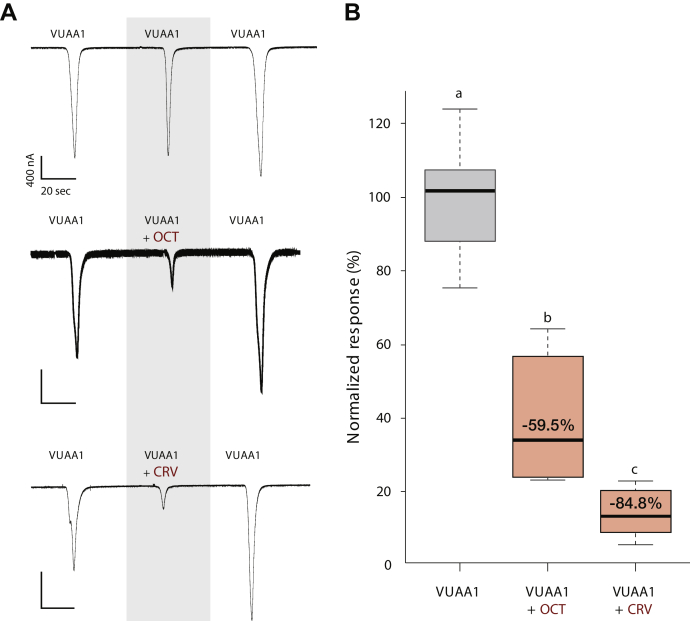
**Octadienal and carvacrol inhibit VUAA1-induced ORco function in frog oocytes.***A*, representative current traces of oocytes expressing AaegORco following exposure to 200 μM VUAA1 alone or in combination with equimolar concentrations of octadienal or carvacrol. *B*, normalized responses of AaegORco to VUAA1 alone or in combination with octadienal or carvacrol. The inhibition levels were calculated by normalizing the response amplitude elicited by the second stimulation to the average current responses elicited by the first and last VUAA1 stimulations shown in *A*. Odorant effects were statistically significant (one-way ANOVA, Df = 2; F = 40.3; *p* < 0.0001) followed by Tukey’s post hoc test (VUAA1 *versus* CRV: *p* < 0.001; VUAA1 *versus* OCT: *p* = 0.001; VUAA1 *versus* OCT *versus* CRV: *p* = 0.001). Mean normalized current responses ± SEM; VUAA1: n = 7; OCT and CRV: n = 6.

To confirm the cross-species bioactivity of these compounds in mosquitoes, we tested the activity of the two most potent antagonists, CRV and OCT (Fig. 3), on *Xenopus* oocytes expressing *Ae. aegypti* ORco (AaegORco). Across all treatments, we observed a consistent increase in the third VUAA1-induced response relative to the first and second VUAA1 administrations (Fig. 4*A*). For this reason, the inhibition level (Fig. 4*B*) was calculated by normalizing the response amplitude elicited by the second stimulation, to the average current responses elicited by the first and last VUAA1 stimulations. Although neither of the two compounds elicited currents in water-injected oocyte controls (Fig. S1), OCT and CRV reduced VUAA1-activated currents (Fig. 4*A*) by approximately 60% and 85%, respectively (Fig. 4*B*), in accordance with the cell-based results, where CRV was found to be a more potent inhibitor compared with OCT (Fig. 3).

### Natural VOCs acting as AgamORco antagonists repel *Ae. albopictus* mosquitoes

The five AgamORco antagonists identified in the cell-based screening assay were subsequently tested for repellence activity against laboratory populations of an aggressive mosquito species, the Asian tiger mosquito, *Ae. albopictus*. Repellency was evaluated by the reduction in the number of mosquito landings on an exposed portion of a human hand. The widely used repellent DEET (45) and the strong mosquito repellent IPC (44, 46), previously characterized as an AgamORco antagonist (34), were used as standards.

**Figure 5:**
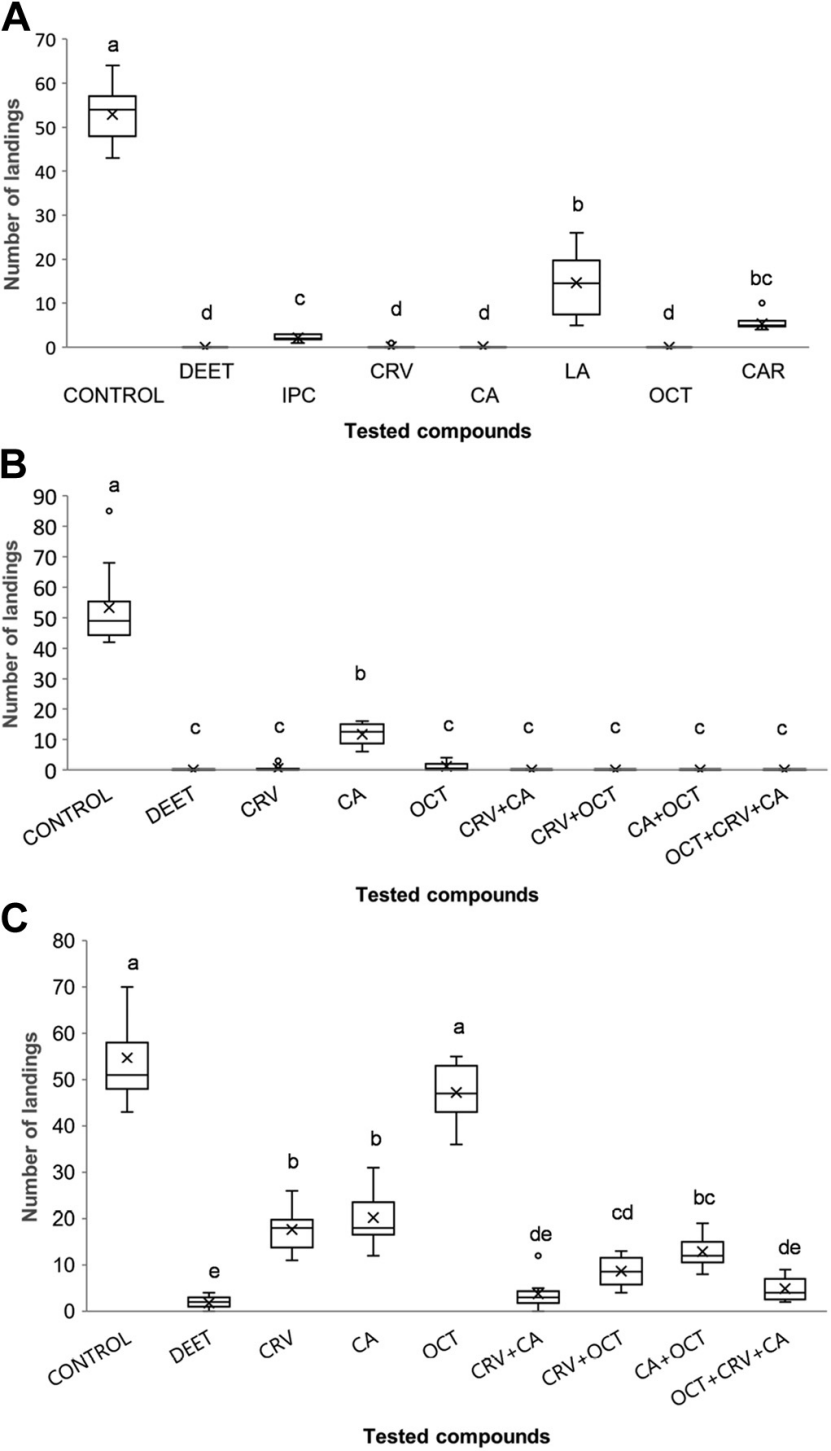
**Repellent activities of ORco antagonists.** Box plots depicting landings distributions of *Ae. albopictus* mosquitoes after exposure to a dose of 0.2 (*A*), 0.04 (*B*), or 0.01 (*C*) μL/cm^2^ (for molar concentrations see also Tables 1 and 2) of tested compounds and selected mixtures, for 5 minutes. On the right half of *B* and *C*, the effects of binary and ternary mixtures are presented. Within each panel, different letters indicate significant differences among tested materials (*p* < 0.05, Mann–Whitney U test with Bonferroni correction). CA, cumin alcohol; CRV, carvacrol; DEET, *N,N*-diethyl-3-methylbenzamide; OCT, (2*E*,4*E*)-2,4-octadienal.

The bioassays (see Table S2 for quantifications of landing numbers) showed that, at the highest tested dose (0.2 μl/cm^2^, 1–1.4 μmole/cm^2^), all compounds significantly reduced mosquito landing counts relative to the solvent controls (Fig. 5*A* and Table 1). At a dose of 0.04 μl/cm^2^ (210–280 nmole/cm^2^), the strongest ORco antagonists, CRV and OCT, displayed repellent activities comparable to that of DEET, whereas the activity of CA was noticeably lower (Fig. 5*B* and Table 1). At the lowest tested dose (0.01 μl/cm^2^, 52–70 nmole/cm^2^), all compounds were found to display repellent activities weaker than that of DEET, with OCT eliciting the lowest repellency of all (Fig. 5*C* and Table 1).

**Table 1:** Repellence indices of tested compounds in human hand landing assays

Compound name (abbreviation)	Structure	Molecular weight	μL equivalent per cm^2^	nmole per cm^2^	Mean % repellency	DEET Pr(<[t])
*N,N*-Diethyl-3-methylbenzamide (DEET)		191.3	0.2	1042	100 ± 0	
			0.04	208	100 ± 0	
			0.01	52	96.7 ± 2.6	
Carvacrol (CRV)		150.2	0.2	1300	99.8 ± 0.7	0.37737
			0.04	260	99.1 ± 2	0.23994
			0.01	65	67.8 ± 9	6.9E-16
Cumin alcohol (CA)		150.2	0.2	1300	99.8 ± 0.7	0.04276
			0.04	260	78 ± 7	1.3E-06
			0.01	65	63.2 ± 11.8	1.3E-14
Isopropyl cinnamate (IPC)		190.2	0.2	1070	96 ± 1.6	0.04835
			0.04	214	-	-
			0.01	54	-	-
Linalyl acetate (LA)		196.3	0.2	920	72.3 ± 15.6	0.00091
			0.04	184	-	-
			0.01	46	-	-
(2*E*,4*E*)-2,4-Octadienal (OCT)		124.2	0.2	1410	100 ± 0	-
			0.04	280	97.9 ± 2.7	0.06309
			0.01	70	13.8 ± 12.8	8.6E-25
(1*S*)-3-Carene (CAR)		136.2	0.2	1270	89.8 ± 4.3	0.00181
			0.04	254	-	-
			0.01	64	-	-

All tests were carried out over a period of 5 min. Dichloromethane was used as a control and the number of landings for the experiments involving compound equivalent doses of 0.2, 0.04, and 0.01 μl/cm^2^ are presented in Table S2. Statistically significant differences, between antagonists and DEET control, are those with Pr(<[0.05]).

-, not examined.

### Binary mixtures of ORco antagonists are more active than single compounds

The three compounds with the strongest repelling indices (Table 1) were subsequently tested in the bioassay as binary and ternary mixtures, using two different doses, medium and low (Fig. 5, *B*–*C*, respectively, and Tables 2 and S2).

**Table 2:** Low doses of compound mixtures are more active repellents than single ones

Compound	μL equivalent of each compound per cm^2^	nmole of each compound per cm^2^	Mean % repellency	DEET Pr(<[t])	Single compound Pr(<[t])		
DEET	0.01	52	96.7 ± 2.6				
CRV	0.01	65	67.8 ± 9.0	6.9E-16			
CA	0.01	65	63.2 ± 11.8	1.3E-14			
OCT	0.01	70	13.8 ± 12.8	8.6E-25			
CRV+CA	0.005	32.5 + 32.5	93.1 ± 6.8	0.02971	**CRV**	**CA**	
					1.8E-05	3.7E-05	
CRV+OCT	0.005	32.5 + 35	84.2 ± 6.6	1.3E-09	**CRV**	**OCT**	
					0.00087	3.9E-09	
CA+OCT	0.005	32.5 + 35	76.5 ± 6.9	8.0E-14	**CA**	**OCT**	
					0.02423	8.3E-08	
CRV+CA+OCT	0.0033	21.7 + 21.7 + 23.3	91.1 ± 5.2	0.00019	**CRV**	**CA**	**OCT**
					4.3E-05	9.6E-05	4.5E-09

All tests were carried out over a period of 5 min. Dichloromethane was used as a control and the numbers of landings for the experiments involving compound equivalent doses of 0.2, 0.04, and 0.01 μl/cm^2^ are presented in Table S2. Statistically significant differences, between antagonists and DEET control, are those with Pr(<[0.05]).

CA, cumin alcohol; CRV, carvacrol; DEET, *N,N*-diethyl-3-methylbenzamide; OCT, (2*E*,4*E*)-2,4-octadienal.

At the lowest antagonist doses examined (0.01 μl/cm^2^), equivolume binary mixtures consisting of 0.005 μl/cm^2^ each of CRV (32.5 nmole/cm^2^) and CA or OCT (32.5 and 35 nmole/cm^2^, respectively) reduced mosquito landing rates by 93.1% (CRV+CA), 84.2% (CRV+OCT), and 76.5% (CA+OCT), respectively. These landing rates were significantly lower than those elicited by each single compound at a dose 0.01 μl/cm^2^ (67.8%, 63.2%, and 13.8% for CRV, CA, and OCT, respectively; Fig. 5*C* and Tables 2 and S2). On the other hand, a ternary mixture, consisting of 0.0033 μl/cm^2^ each of CRV (21.7 nmole/cm^2^), CA (21.7 nmole/cm^2^), and OCT (23.3 nmole/cm^2^) displayed repellence activity of 91.1% against *Ae. albopictus*, comparable with that of the CRV+CA binary mixture at the same total antagonist amount of 65 nmole/cm^2^ (93.1%).

### Binding competition assays reveal possible mechanism for the enhanced activity of binary mixtures

A possible explanation for the increased repellent activity of binary antagonist mixtures is that it may be caused by their combined interactions within the ORcoRAM2 binding site (47) or with additional, distinct binding sites. Such interactions could impose enhanced conformational rearrangements in ORco, steric hindrance in the agonist binding site, and enhanced inhibition of ORco function. Moreover, given the small size of at least some of the identified antagonists relative to OAs, it is also possible that a single binding site, *e.g.*, the one to which the ORco agonist binds, could accommodate the binding of two different antagonists that act in an additive fashion thus causing a higher degree of ORco inhibition relative to the single ones.

To distinguish between competitive (orthosteric) and non-competitive (allosteric) interactions, we carried out binding competition experiments. These assays involved antagonist and agonist dose-dependent binding to AgamORco that could distinguish between competitive and noncompetitive binding of the examined antagonists relative to the binding site of the ORco agonist ORcoRAM2 (47).

**Figure 6:**
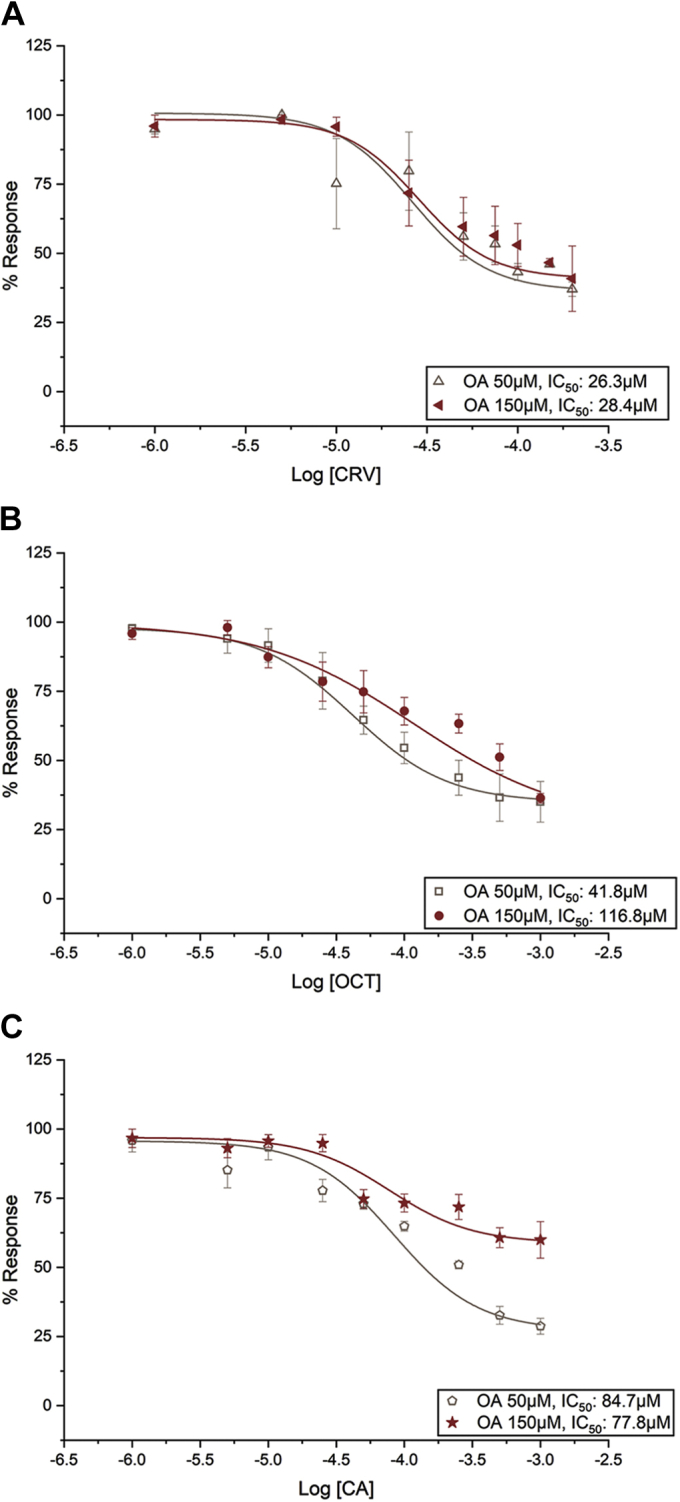
**ORco competition assays as dose-dependent antagonist effect.***A*, the IC_50_ values determined for carvacrol (CRV) in the presence of 50 and 150 μM ORcoRAM2 were 26.3 μM (pIC_50_: 4.58001 ± 0.21732, R^2^: 0.99998) and 28.4 μM (pIC_50_: 4.54608, R^2^: 0.99747), respectively. There is no significant change in the IC_50_ as the ORco agonist (OA) concentration increases (IC_50_ of CRV with 100 μM OA is 23.4 μM), expected when compounds bind to different binding sites, with allosteric antagonistic effect. *B*, the IC_50_ values determined for octadienal (OCT) in the presence of 50 and 150 μM ORcoRAM2 were 41.8 μM (pIC_50_: 4.37887 ± 0.03444, R^2^: 0.99999) and 116.8 μM (pIC_50_: 3.93263 ± 0.26793, R^2^: 0.99973), respectively. There is a dextral shift of the curve and a concomitant increase of the IC_50_ as the OA concentration is increased, expected when both compounds compete for the same binding site. *C*, the IC_50_ values determined for cumin alcohol (CA) in the presence of 50 and 150 μM ORcoRAM2 were 84.7 μM (pIC_50_: 4.07226 ± 0.23144, R^2^: 0.99998) and 77.8 μM (pIC_50_: 4.10898 ± 0.17809, R^2^: 0.99984), respectively. Error bars indicate mean ± SE. Data points were normalized to the maximum value (set at 100%).

The antagonist dose–response experiments were performed in the presence of 50 and 150 μM OA. For CRV, with IC_50_ values of 26.3 and 28.4 μM in the presence of 50 and 150 μM OA, respectively (Fig. 6*A* and Table S3), the results suggest a non-competitive (allosteric) antagonist of ORcoRAM2. In contrast, with decreasing potency in the presence of increasing OA amounts (IC_50_ of 41.8 and 116.8 μM in the presence of 50 and 150 μM of ORcoRam2, respectively; Fig. 6*B* and Table S3), OCT behaved as a competitive (orthosteric) inhibitor of ORcoRAM2. Cumin alcohol (CA), on the other hand, also behaved as an allosteric inhibitor of ORco function as its IC_50_ values in the presence of 50 and 150 μM ORcoRAM2 are maintained at similar levels (84.7 and 77.8 μM, respectively; Fig. 6*C* and Table S3). However, relative to CRV, CA is a less efficacious antagonist, as its ability to antagonize the effect of 150 μM ORcoRAM2 is reduced significantly.

**Figure 7:**
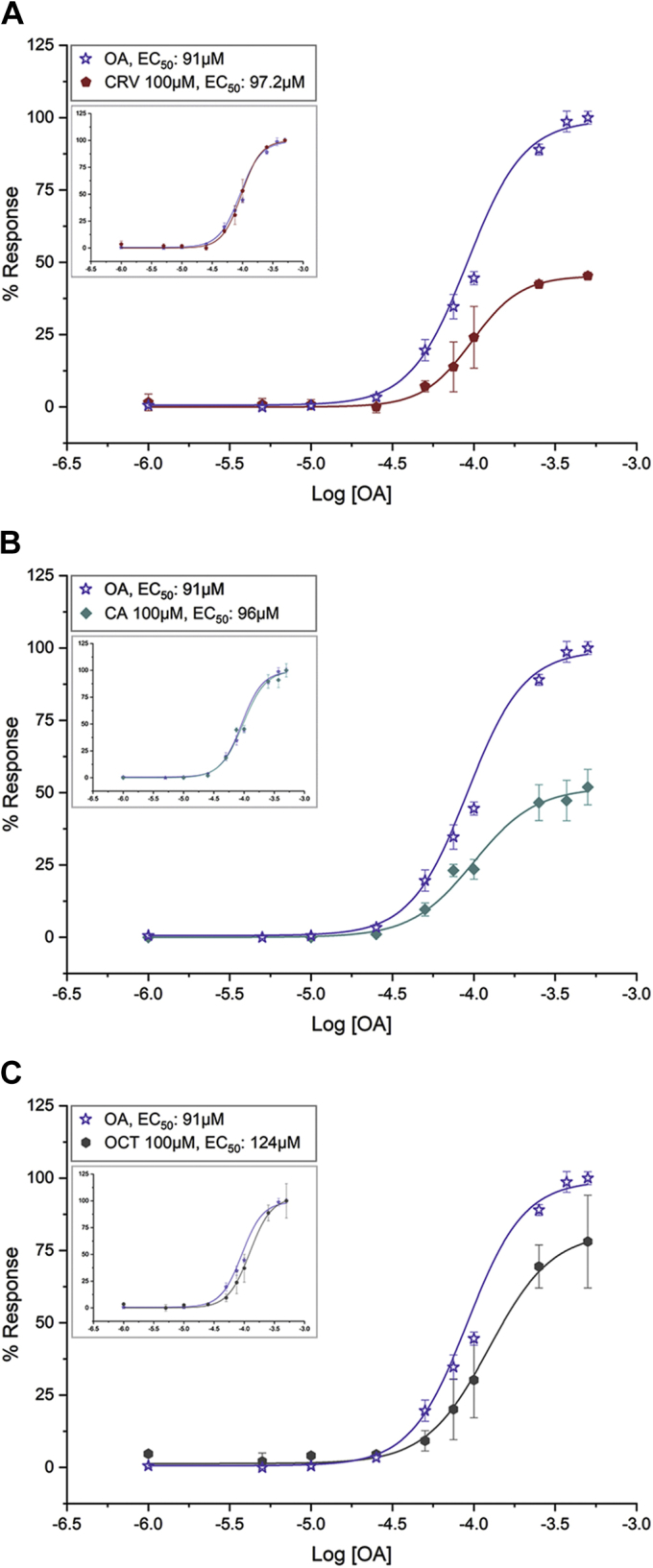
**ORco competition assays as dose-dependent agonist effect.** ORcoRAM2 (OA) EC_50_ in the absence of antagonists depicted in all panels is 91 μM (pEC_50_: 4.0409 ± 0.71927, R^2^:0.99741). *A*, the OA EC_50_ value in the presence of 100 μM carvacrol (CRV) was 97.2 μM (pEC_50_: 4.01241 ± 0.56797, R^2^: 0.97787). There is no noticeable shift in the EC_50_ concentration in the absence or presence of CRV (91 and 97.2 μM, respectively; *inset*: graphs after normalization of maximum responses for both curves to 100%), expected when compounds bind to different binding sites, with allosteric antagonistic effect. *B*, the EC_50_ value of ORcoRAM2 in the presence of 100 μM cumin alcohol (CA) was 96 μM (pEC_50_: 4.00222 ± 0.94917, R^2^: 0.98670). There is no noticeable shift in the EC_50_ concentration in the absence or presence of CA (91 and 96 μM, respectively; *inset*: graphs as per *A*), expected when compounds bind to different binding sites, with a non-competitive effect. *C*, the EC_50_ value of ORco agonist in the presence of 100 μM octadienal (OCT) was 124 μM (pEC_50_: 3.90655 ± 0.65211, R^2^: 0.99604). There is a dextral shift of the curve and an increase of the EC_50_ in the presence of octadienal (from 91 to 124 μM; *inset*: graphs as per *A* and *B*), expected when both compounds compete for the same binding site.

Additional experimentation involving agonist dose–response measurements, in the absence or presence of the specific antagonists (Fig. 7 and Table S4), confirmed these conclusions. Specifically, the ORcoRAM2 dose–response curves in the presence of 100 μM CRV (Fig. 7*A* and Table S4) or 100 μM CA (Fig. 7*B* and Table S4) revealed reduced responses at 45% and 52% with very similar EC_50_ values of 96 and 97.2 μM, respectively, relative to the maximal (100%) ORco response with an EC_50_ value of 91 μM in the absence of antagonist. In contrast, with a smaller reduction in OA-induced ORco activity at 78% in the presence of 100 μM OCT, a clear rightward shift of the OA dose–response curve was observed, with the EC_50_ value increasing to 124 μM (Fig. 7*C* and Table S4).

### Enhanced ORco inhibition by antagonist blends: additive or synergistic effects?

**Figure 8:**
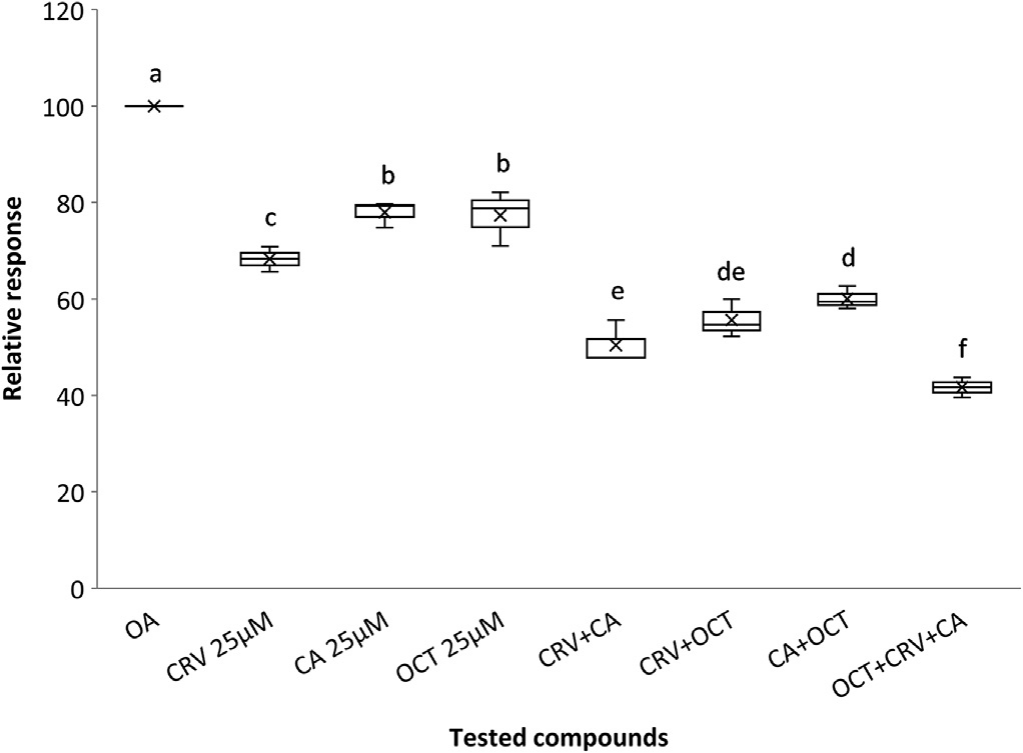
**ORco functional assays of antagonist mixtures.** Box plots depicting relative AgamORco luminescence responses induced by 100 μM OA (ORcoRAM2) in the absence (OA) or presence of low concentrations of antagonists and mixtures thereof, consisting of 25 μM each. The examined blends display enhanced mosquito repellent activities that are probably the result of additive rather than a synergistic effect as depicted here. Different letters (a, b, etc.) indicate statistically significant differences between tested compounds (one-way ANOVA, Df = 7; F = 88.4; *p*= 1.36E-11; followed by Mann–Whitney U test with Bonferroni correction, *p* < 0.05). CA, cumin alcohol; CRV, carvacrol; OA, ORco agonist; OCT, (2*E*,4*E*)-2,4-octadienal.

To address the question of whether the enhanced repellence activities of antagonist blends observed in the behavioral assays (Fig. 5) are due to additive or synergistic structural and consequent functional effects of the specific compounds on ORco, we undertook quantitative assessments of OA binding in the presence of low doses of the specific antagonists and their mixtures. These experiments, whose results are shown in Figure 8 and Table 3, revealed interesting binding and functional correlations for the tested ORco antagonists. First, in all examined cases of antagonist blends, binary or ternary ones, which contained antagonists at a concentration of 25 μM each, the resultant reduction in OA-induced ORco function (cellular luminescence) has been significantly more pronounced relative to that effected by 25 μM of each antagonist alone (Table 3). Therefore, the binding of a specific antagonist to ORco did not prevent the binding of and further functional inhibition of ORco function by another antagonist, orthosteric or allosteric. The same experiments, however, also revealed that the ORco activity reductions effected by the examined antagonist mixtures in no case exceeded the predicted additive sum of inhibition exerted by each individual constituent added at the same concentration of 25 μM (Table 3). Thus, in all examined cases of binary mixtures, the observed reduction in cellular luminescence has been somewhat smaller than the sum of inhibitory effects exerted by each individual antagonist. This was even more pronounced in the case of the CRV + OCT and the ternary mixture, where the observed combined reductions in luminescence were significantly smaller than the predicted sums of individual antagonist effects. These results appear to exclude the possibility of synergistic effects between the examined orthosteric and allosteric ORco antagonists. Instead, they suggest that the enhanced repellence activities of antagonist blends are likely due to the additive effects of the antagonist combinations. Moreover, it appears that the binding of one antagonist may interfere, to a certain extent, with the binding of another one. An additional interesting result of these experiments that correlates with the *Ae. albopictus* repellency findings (Fig. 5) has been that, in all cases, the reductions in OA-induced activity of ORco effected by the blends were higher than those effected by each individual blend component when the latter was added at a concentration equal to the total compound concentration of each blend (50 μM for the binary blends, 75 μM for the ternary ones; Table 3).

**Table 3:** Functional effects of low concentrations of antagonist mixtures on ORco function

Compound/mixtures	Compound conc. (μM)	% Inhibition (±SD)	Predicted additive sum of % inhibition	Blends *versus* single compound at 25 μM Pr(<[t])			Predicted *versus* actual blend inhibition Pr(<[t])	Blend *versus* single compound at 50/75 μM Pr(<[t])^a^		
OA	100									
CRV	+25	31.75 ± 1.21								
	+50	43.50 ± 2.51								
	+75	47.20 ± 1.82								
CA	+25	22.08 ± 0.78								
	+50	27.46 ± 2.90								
	+75	39.41 ± 3.09								
OCT	+25	22.70 ± 1.68								
	+50	30.68 ± 3.58								
	+75	39.93 ± 2.29								
CRV+CA	+25+25	49.61 ± 4.46	53.83	CRV	CA		0.232882	CRV	CA	
				0.00001	0.000001			0.023823	0.001242	
CRV+OCT	+25+25	44.38 ± 3.15	54.45	CRV	OCT		0.021612	CRV	OCT	
				0.000267	0.000004			0.708954	0.000446	
CA+OCT	+25+25	39.96 ± 1.58	44.78	CA	OCT		0.391426	CA	OCT	
				0.000447	0.00025			0.051099	0.021817	
CRV+CA+OCT	+25+25+25	58.34 ± 2.96	76.53	CRV	CA	OCT	0.001936	CRV	CA	OCT
				5.75E-09	8.61E-09	1.24E-09		0.000065	0.000004	0.000005

Inhibition of relative luminescence of ORco responses upon OA induction (100 μM) in the presence of low concentrations of selected antagonists and mixtures thereof (three repeat experiments, each in triplicate). Statistically significant differences, between mixtures and single compounds, are considered those with Pr(<[0.05]).

+, in addition to 100 μM OA; CA, cumin alcohol; CRV, carvacrol; DEET, *N,N*-diethyl-3-methylbenzamide; OCT, (2*E*,4*E*)-2,4-octadienal.

a Fifty μM single compound concentration for two-compound mixtures, 75 μM single compound concentration for three-compound mixtures.

## Discussion

OBPs and ORs expressed predominantly in female mosquitoes are known to constitute promising targets for the discovery of molecules capable of altering the odor-sensing capacity and odor-evoked behaviors of mosquitoes (48). Our previous work has shown that some strong mosquito repellents of natural origin (33) act as ORco antagonists (34). Moreover, we have shown that ORco-specific synthetic agonists, such as VUAA1 and OrcoRAM2, activate ORx/ORco channels in cultured insect cells (34) and also act as positive allosteric modulators of odorant receptor function (35). Thus, ORco is a rational target for molecules that may function as modulators of peripheral olfactory functions in mosquitoes and, probably, other insect species as well. Consequently, the employment of screening platforms that exploit the capacity of ORco to form functional homomeric ion channels in cultured cells and the identification of specific ORco agonists or antagonists, may result in the discovery of natural or synthetic modulators of olfaction-dependent mosquito behaviors. Such modulators may be either “anosmia”-inducing factors or olfactory enhancers.

In this report, we present an integrated study that includes the design and use of a convenient screening platform that allows identification of ORco functional modulators from collections of metabolites of natural or synthetic origin, as well as examination of pharmacological and functional aspects of identified modulators. Specifically, the screening of 50 natural metabolites, whose common property has been their volatility, resulted in the detection of three novel AgamORco antagonists, LA, OCT, and CAR (Figs. 2 and 3). Two additional compounds, CRV and CA, which were previously shown to inhibit the function of AgamORco and several AgamORx/ORco receptors (34), were confirmed here to be AgamORco antagonists and further characterized pharmacologically (Figure 2, Figure 3, Figure 4, 6 and 7). On the other hand, the specific screening initiative did not reveal the presence of VOCs capable of fulfilling the agonist criteria we defined at the outset of this study, *i.e.*, the magnitude of primary addition responses approaching those of the known OA, OrcoRAM2. We note, however, the presence of several compounds that effect low primary luminescence responses. Although these potential agonists were not analyzed further in the context of this report, they await further characterization in future studies.

Of interest, the identified antagonists are not characterized by the presence of a single functional group. Thus, CRV and CA are aromatic ligands decorated with electrophilic functionalities, whereas LA and OCT are similarly decorated aliphatic compounds. On the other hand, CAR is a bicyclic nonpolar molecule. As discussed below, such structural differentiation may relate to the nature of the binding sites of these compounds on ORco.

Our earlier findings concerning the bioactivities of CRV and CA, now confirmed to be AgamORco antagonists, which were shown to repel effectively *An. gambiae* and *Culex* spp mosquitoes (33), raised the question of whether these as well as the new antagonists identified in this study were also active against *Aedes*, the third major mosquito genus of the Culicidae family, which comprises multiple hematopagous species and infectious disease vectors. The initial testing of two selected antagonists, CRV and OCT, in *X. laevis* oocytes expressing the ORco subunit of *Ae. aegypti* revealed a significant inhibition of AaegORco function (Fig. 4). In addition, all identified antagonists were shown to repel *Ae. albopictus* mosquitoes to various degrees (Fig. 5 and Table 1). The combined results constitute proof of principle for the notion that AgamORco antagonists are efficient blocking agents of olfactory function in multiple mosquito genera. Therefore, the search for new compounds capable of interfering with mosquito olfactory functions by screening VOC collections for ORco, as opposed to multiple ORx-specific antagonist activities, is both relevant and feasible.

A recent study involving the functional screening, in *X. laevis* oocytes expressing AgamOrco, of a small collection of commercially available natural compounds selected through machine learning methodologies, identified two AgamOrco antagonists, which inhibited odorant responses in electroantennogram and single sensillum recordings of adult *Drosophila melanogaster* antennae, and inhibited odorant-directed behaviors in larvae of the same species (49). Of interest, this study, whose results are concordant with ours with respect to the cross-genus bioactivities of AgamORco antagonists, identified linalyl formate, a compound with a structure nearly identical to that of LA, as one of the two AgamORco antagonists that inhibited odorant-directed behaviors in *Drosophila* larvae.

The combined findings on the physiological and behavioral effects of AgamORco antagonists on different dipteran species are obviously due to the very high phylogenetic conservation of ORco among insect species (10, 11, 12, 13, 14). In this regard, we note that the notion that agonists capable of activating constitutively the common subunit of odorant receptors should cause olfactory confusion has also been proposed in the past (25). However, relevant behavioral experimentation to confirm this notion has not been presented.

An obvious aspect that needs to be explored further concerns the molecular mechanism underlying the behavioral effects of the identified AgamORco antagonists on the targeted mosquitoes. Although explanations involving ORco-independent pathways may be invoked to explain the behavioral changes induced in mosquitoes exposed to these volatile antagonists, our results are consistent with the hypothesis that the observed effects are due to the functional inhibition of the olfactory apparatus caused by their direct binding to the obligatory ORco subunit of odorant receptors. Thus, the inhibitors of ORco homomeric channels formed in cultured cells apparently become common intraspecific inhibitors of essentially all ORx/ORco heteromeric receptors *in vivo*, in a way analogous to but much broader than the recently proposed intraspecific inhibitors of heteromeric receptors (50). Based on the proposed ability of the identified ORco-targeting VOCs to inhibit the function of multiple ORs, we consider it likely that they cause anosmia-like effects to the targeted mosquitoes. The proposed mode of action for the identified bioactive VOCs is thus distinct from the receptor-independent function of DEET, which was recently shown to “repel” mosquitoes and other insects, at least in part, via its association with volatile receptor ligands acting as attractants, thereby reducing their volatility and effective concentrations thus masking their presence in the mosquito’s environment (51).

An additional noticeable finding of the study reported here has been the enhanced repellent action of binary combinations of ORco antagonists on the behavior of *Ae. albopictus* adults. Such effects had been noted previously in our studies on laboratory and field populations of *An. gambiae* and *Culex* spp (33). The CA+CRV mixture, in particular, which displayed repellent activity against *Ae. albopictus* very similar to DEET (landing inhibition of 93.1% *versus* 96.7%), was previously shown to also repel *An. gambiae* and *Culex* spp mosquitoes in the field more effectively than DEET (33). The enhanced bioactivities of binary mixtures of chemically diverse AgamORco antagonists raised the question of the possible relevance of antagonist binding sites to the observed repellence behaviors. The binding competition assays for CRV and CA against ORcoRAM2 (Fig. 6, *A*–*C*) suggested that the two compounds, which maintain very similar IC_50_ values in the presence of 50 or 150 μM (as well as 100 μM; Fig. 3*A* and Table S3) OA, bind to sites different from the one that ORcoRAM2 binds and are therefore noncompetitive, allosteric antagonists of the OA-binding site on the AgamORco tetramer (47). The OA dose–response curves in the presence of each of the two antagonists (Fig. 7, *A*–*B* and Table S4) confirmed this conclusion.

The enhanced performance of the CRV+CA mixture in the mosquito landing inhibition assays relative to CRV or CA alone (Table 2) further suggests separate CRV and CA binding sites despite the apparent structural similarities between these two compounds. The alternative possibility that these two compounds bind to different sections of a common allosteric binding pocket cannot be excluded without further experimentation. In contrast, the binding competition assays for OCT (Figs. 6*B* and 7*C*) suggest that this compound is a competitive, orthosteric antagonist of AgamORco with respect to its agonist (ORcoRAM2) binding site. Accordingly, we are attributing the enhanced inhibitory effects of the CRV+OCT and CA+OCT mixtures, relative to CRV, CA, or OCT alone, to the simultaneous binding of an orthosteric and an allosteric antagonist on *Ae. albopictus* ORco (AalbORco). The additional OA binding competition and consequent ORco functional assays in the simultaneous presence of low concentrations of two or three antagonists (Fig. 8), whose individual presence caused only minor inhibitory effects on ORco function, suggested that the augmented inhibition of the antagonist blends is probably the result of additive rather than synergistic inhibitory effects. To our knowledge, this constitutes the first evidence of such type of inhibition on a constituent subunit of insect odorant receptors.

Although the antagonist binding competition experiments and associated inhibitory effects on ORco activity resulting from their concomitant presence provide clues related to the nature of their binding sites relative to the postulated one for ORcoRAM2 (47), the precise nature of the binding sites, particularly for CRV and CA, remains to be determined. Molecular dynamics and molecular docking studies are currently in progress in an effort to identify candidate binding sites in the recently resolved homotetrameric complex of the ORco channel (47). Ultimate proof for our hypotheses concerning the specific antagonist-binding sites will have to await the undertaking and evaluation of mutagenesis studies.

The cross-species bioactivities of compounds with mosquito repelling activities discovered through the AgamORco VOC screen, which, owing to the high conservation of ORco across phylogeny, are also capable of repelling, thus offering biting protection from other insects and arachnids such as *Lutzomyia longipalpis* sandflies and *Ixodes ricinus* ticks (52), may raise concerns regarding their environmental safety. In this respect, it should be stressed that such repellent compounds pose no danger to the environment, as they are destined to be used on a limited scale, either for personal protection or as spatial repellents. Moreover, the demonstrated superiority of antagonist blends over single ones, at least for the antagonists tested here, provides an added consideration for health and general environmental safety, as the lower component concentrations in the binary and ternary mixtures are likely to be less toxic than the higher concentrations of individual antagonists needed to achieve equivalent behavioral effects.

## Experimental procedures

### Mosquitoes

Adult *Ae. albopictus* mosquitoes used for the repellency assays were from a laboratory colony maintained at 25 ± 2 °C, 80% relative humidity, and 16/8-h light/dark photoperiod at the Benaki Phytopathological Institute, Kifissia, Greece (53). Plastic beakers with 100 ml water and strips of moistened filter paper were inserted in the cages for oviposition. The eggs were kept damp for a few days and then placed in enamel pans for hatching. The larvae were reared in tap water–filled cylindrical enamel pans, approximately 400 larvae per pan, and were fed *ad libitum* with powdered fish food (JBL Novo Tom 10% Artemia) until the emergence of adults. Adult mosquitoes were collected periodically with a mouth aspirator and transferred to a rearing cage. Females were fed with fresh chicken blood using a Hemotek blood feeding system (54).

### Chemicals

ORco agonists, repellents, and the 50 VOCs analyzed in the current study are presented in Table S1. Carvacrol (CRV), linalyl acetate (LA), (2*E*,4*E*)-2,4-octadienal (OCT), and (1S)-3-carene (CAR) were purchased from Sigma Aldrich; isopropyl cinnamate (IPC) from Alfa Aesar; cumin alcohol (CA) from Acros Organics; *N*-(4-ethylphenyl)-2-{[4-ethyl-5-(3-pyridinyl)-4H-1,2,4-triazol-3-yl]thio}acetamide (ORco Receptor Agonist Molecule 2, ORcoRAM2) from Asinex Corporation and Vitas M Chemical Ltd; *N*-(4-ethylphenyl)-2-{[4-ethyl-5-(3-pyridinyl)-4H-1,2,4-triazol-3-yl]thio}acetamide (VUAA1) from Innovapharm Ltd; *N,N-*diethyl-3-methylbenzamide (DEET) from Sigma-Aldrich; and coelenterazine from Biosynth. Initial stock solutions for ORcoRAM2 and VUAA1 were prepared as needed in dimethyl sulfoxide (DMSO) and stored at −20 °C, whereas initial stocks of VOCs and coelenterazine were prepared in ethanol as needed and stored at −20 °C. For the insect cell–based screening assay, working solutions were prepared in modified Ringer's buffer (25 mM NaCl, 190 mM KCl, 3 mM CaCl_2_, 3 mM MgCl_2_, 20 mM Hepes, 22.5 mM glucose, pH 6.5; 35), so that the final DMSO concentrations did not exceed the range of 0.2% to 0.35%.

### Transformation of Bm5 cells for AgamORco and Photina expression and Ca^2+^ influx assays

The screening platform shown in Figure 1 was employed as a tool for discovery of new compounds capable of modulating mosquito ORco activity and olfaction-mediated behaviors. It consists of lepidopteran cultured cells (*Bombyx mori* Bm5; (55)) expressing constitutively AgamORco, which forms a ligand-gated ion channel (34, 35), and Photina (56), a reporter photoprotein activated by Ca^2+^ ions entering the cells upon activation of the ORco channel. Briefly, Bm5 cells were stably transformed to express the cDNAs for AgamORco and the reporter photoprotein Photina from high-expression-level pEIA plasmid vectors (57, 58, 59, 60) as described (21, 34). Cell lines were maintained at 28 °C and grown in IPL-41 insect cell culture medium (Genaxxon Bioscience GmbH) supplemented with 10% fetal bovine serum (Biosera) in the presence of 10 μg/ml puromycin. Ligand binding to the ORco channel and subsequent functional effects were monitored via luminescence emission, using Photina as Ca^2+^ influx biosensor as described (34, 35). Specifically, cells were resuspended in modified Ringer's buffer, seeded in a white 96-well plate (200,000–300,000 cells/well), and incubated with 5 μM coelenterazine for 2 h at room temperature in the dark. Baseline and maximum luminescence outputs, obtained by the addition of buffer and 1% Triton-X100, respectively, were recorded in an Infinite M200 microplate reader (Tecan) at 4-s intervals for up to 20 s. The cells were subjected to two cycles of compound additions. Initially, a tested compound was added at 100 μM and the ORco channel response was monitored. Cells were allowed to return to baseline luminescence and the addition of 100 μM of the ion channel–activating OA followed, measuring the secondary effect of ligand binding in terms of luminescence emission (4-s intervals for 80 s). Initial luminescence data were acquired using i-Control 1.3 software by Tecan. Relative luminescence values were normalized by considering ORco agonist luminescent response as the maximal (100%) receptor response for each set of experiments. Each independent experiment was run in triplicate and repeated at least three times.

### Binding assays

Solvent or identified antagonists at concentrations ranging from 1 μM to 1 mM were added to cells expressing AgamORco and Photina, previously seeded in wells of white 96-well plates and incubated with 5 μM coelenterazine as described above, and the induced luminescence, if any, was measured. This was followed by addition of OA (ORcoRAM2) to each well at a final concentration of 50, 100, or 150 μM, depending on the type of dose-dependent assay. For initial determination of existing antagonist activities, 100 μM of OA was used. To evaluate the type of binding on ORco, orthosteric or allosteric relative to the OA-binding site, antagonist dose-dependent inhibition assays were carried out in the presence of 50 and 150 μM OA. Confirmation of the conclusions of these competition experiments, in relation to the nature of the ligand-binding sites, was obtained by OA dose–response assays (1–500 μM concentrations) in the presence of solvent or 100 μM of each tested antagonist and EC_50_ determination under each condition. Curve fitting and EC_50_/IC_50_ value calculations were carried out using OriginPro 8 software by OriginLab Corporation. Dose–response curves were plotted by fitting the normalized data into the equation $y=A_{1}+\frac{A_{2}-A_{1}}{1+10^{\left(LogEC50-x\right)p}}$, where A_1_ and A_2_ are the bottom and top asymptotes, respectively, *p* is the Hillslope, *y* is the percent response at a given concentration, and *x* is logarithm of ligand concentration. Statistically significant differences between IC_50_ values of antagonists in the presence of 50 μM OA relative to the IC_50_ values in the presence of the higher OA concentrations (100 and 150 μM; Table S3), as well as EC_50_ values in the absence or presence of the tested antagonists (Table S4), were evaluated with two-sample *t* test, assuming equal variances. Cell-based functional assays of antagonist binary or ternary mixtures were also carried out at low antagonist concentrations. Blends of antagonists, at 25 μM each, were employed to determine their inhibitory effects on ORco responses induced by 100 μM OA. Antagonists alone at 25, 50, and 75 μM concentrations, in conjunction with 100 μM OA, were also used as single antagonist inhibition controls, whereas OA-induced luminescence values in the absence of antagonists provided the maximal (100%) receptor responses. Statistically significant differences among samples were determined with one-way ANOVA, followed by Mann–Whitney U tests with Bonferroni correction (61; Fig. 8), whereas pair-wise analyses between mixtures and single compounds (Table 3) were carried out using two-sample *t* test, assuming equal variances. Each independent experiment was run in triplicate and repeated at least three times.

### Expression of AaegORco in *X. laevis* oocytes and electrophysiological recordings

*In vitro* transcription and two-microelectrode voltage-clamp electrophysiological recordings were performed as described (62). Briefly, ORco of *Ae. aegypti* (AaegORco; GenBank: BK006142.1) was synthesized using the mMESSAGE mMACHINE SP6 kit (ThermoFisher Scientific) from the linearized pSP64tRFA expression vector. The harvested *X. laevis* oocytes were manually separated from the ovaries prior to collagenase treatment (8 mg/ml, 30 min, 18 °C) in order to remove the follicular layer. Stage V–VI oocytes were rinsed in washing solution (96 mM NaCl, 2 mM KCl, 5 mM MgCl_2_, and 5 mM Hepes, pH 7.6) and microinjected with a mixture of 1 μl AaegOrco (3 μg/μl) and 2 μl of double-distilled water. Injected oocytes were incubated at 18 °C for 3 days in Ringer’s solution (96 mM NaCl, 2 mM KCl, 5 mM MgCl_2_, 0.8 mM CaCl_2_, and 5 mM Hepes, pH 7.6) supplemented with 5% dialyzed horse serum, 50 μg/ml tetracycline, 100 μg/ml streptomycin, and 550 μg/ml sodium pyruvate. Whole-cell currents were recorded using the two-microelectrode voltage-clamp technique. During recording sessions, the holding potential was maintained at −80 mV using an OC-725C oocyte clamp (Warner Instruments, LLC). Oocytes were placed in a RC-3Z oocyte recording chamber (Warner Instruments) and exposed for 8 s to 200 μM VUAA1 (Innovapharm Ltd), 2,4-octadienal predominantly trans (Sigma-Aldrich), or carvacrol (Sigma-Aldrich). All compounds were solubilized in 200 μl of DMSO prior to the dilutions in the Ringer’s solution. Currents were allowed to return to baseline between odorant applications. Data acquisition and concentration–response analyses were carried out with a Digidata 1550A and the pCLAMP10 software (Molecular Devices). Statistical significance was evaluated with one-way ANOVA followed by Tukey's post test.

### Repellence bioassays

For the *in vivo* determination of the repellent activity of VOCs, the assessment was based on human hand landing counts (63). The study was conducted using cages (33 × 33 × 33 cm) equipped with a 32 × 32 mesh at one side, each containing one hundred 5- to 10-day-old adult mosquitoes (sex ratio, 1:1) starved for 12 h at 25 ± 2 °C and 70% to 80% relative humidity. A volunteer’s hand covered by a plastic glove with a dorsal side opening measuring 5 × 5 cm was employed for all bioassays. Tested compounds were applied on chromatography paper (Whatman), over a 24 cm^2^ total area, at three doses equivalent to 0.2, 0.04, and 0.01 μl/cm^2^ (1000–1400, 200–280, and 50–70 nmole/cm^2^, respectively, depending on compound molecular mass; see also Table 1) diluted with dichloromethane (DCM). Control experiments with compound-free DCM solvent or DEET treatments (negative and positive controls, respectively) were included as standards. Each treatment was repeated eight times on four human volunteers. Replicate experiments were n = 15 for the solvent- and DEET-treated controls. The effects of tested ORco antagonists on *Ae. albopictus* landings were estimated using the Kruskal–Wallis test (64). When significant differences were detected, Mann–Whitney U tests with Bonferroni correction (61) were carried out for comparison among all samples. Mosquito landings for each treatment were counted over 5-min periods. Landing numbers were converted to repellence indices (RI±SE) using the equation RI = [1−$\frac{\text{T}}{\text{C}}$] × 100, where *C* is the number of landings in control and *T* the number of landings in treatment. Statistically significant differences, between DEET and each mixture with respect to antagonists alone, were also evaluated with two-sample *t* test, assuming equal variances.

### Ethics statement

The laboratory strain of *Ae. albopictus* used in this study was established using mosquito eggs collected from ovitraps from different areas in Greece. The collection areas were public and not privately owned or protected. Mosquito egg collections from the field did not involve endangered or protected animal species. Consequently, the establishment of the laboratory mosquito strain did not require a specific permit. The repellence studies abide by the Declaration of Helsinki principles. The Ethics Committee of Benaki Phytopathological Institute concluded that the current study was implemented in accordance with the Ethics Code for Research.

## Data availability

All data are contained within the manuscript.

## Conflict of interest

The authors declare that they have no conflicts of interest with the contents of this article.
