# Supplementary material for: Volatile allosteric antagonists of mosquito odorant receptors inhibit human-host attraction
Source: J Biol Chem. 2020 Dec 18;296:100172. doi: 10.1074/jbc.RA120.016557 (PMC7948460; doi:10.1074/jbc.RA120.016557)
Supplement: Supplementary file 1 — Tables S1 to S4 and Figure S1 [file mmc1.pdf]

SUPPORTING INFORMATION

Table S-1: Volatile organic compounds (VOCs) of natural origin selected for screening and other compounds used in this study. With the exception of compounds IV and V that were dissolved in DMSO, all compounds were initially dissolved in ethanol. Abbreviations used throughout in the figures or in the text are presented in parentheses.

No	Compound (abbrev)	CAS No	Structure	MW	Source
I	Carvacrol (CRV)	499-75-2	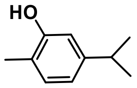	150.2	Plants
II	Isopropyl cinnamate (IPC)	7780-06-5	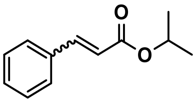	190.2	Plants
III	Cumin alcohol (CA)	536-60-7	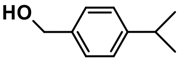	150.2	Plants
IV	ORcoRAM2	618427-06-8	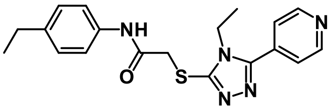	367.5	Synthetic
V	VUAA1	525582-84-7	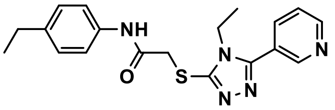	367.5	Synthetic
VI	*N*,*N*-Diethyl-3-methylbenzamide (DEET)	134-62-3	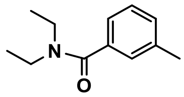	191.3	Synthetic
1	2,5-Dihydrofuran	1708-29-8	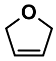	70.1	Plants
2	2-Butanone	78-93-3	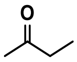	72.1	Bacteria
3	Butyl amine	109-73-9	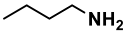	73.1	Plants, bacteria
4	Linalyl acetate (LA)	115-95-7	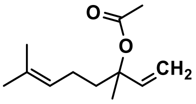	196.3	Plants
5	(*S*)-2-Butanol	4221-99-2	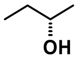	74.1	Bacteria
6	Pyridine	110-86-1	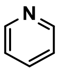	79.1	synthetic
7	Pyrazine	290-37-9	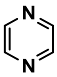	80.1	Bacteria
8	2-Cyclopenten-1-one	930-30-3	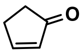	82.1	Plants
9	Thujopsen	470-40-6	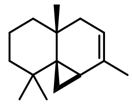	204.4	Plants
10	3,4-Dihydro-2*H*-pyran	110-87-2	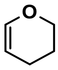	84.1	Synthetic
11	Cyclopropyl methyl ketone	765-43-5	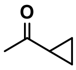	84.1	Synthetic

12	2-Pyrrolidone	616-45-5	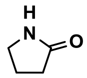	85.1	Spiders
13	2-Pentanone	107-87-9	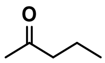	86.1	Plants
14	*N*-Methyl propanamide	1187-58-2	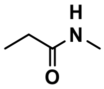	87.1	Algae
15	1,3-Butanediol	107-88-0	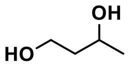	90.1	Bacteria
16	Phenol	108-95-2	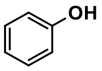	94.1	Bacteria
17	(2E,4E)-2,4-Hexadienal	142-83-6	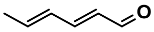	96.1	Insects
18	3-Methyl-2-cyclopenten-1-one	2758-18-1	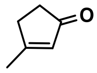	96.1	Bacteria
19	Furfuryl alcohol	98-00-0	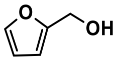	98.1	Bacteria
20	(*E*)-3-Hexen-1-ol	928-97-2	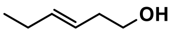	100.2	Plants
21	δ-Valerolactone	542-28-9	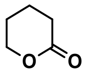	100.1	Plants
22	γ-Valerolactone	108-29-2	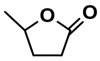	100.1	Bacteria
23	α-Methyl-γ-butyrolactone	1679-47-6	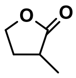	100.1	Fungi
24	2,3-Pentandione	600-14-6	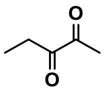	100.1	Yeast
25	Methyl isobutyrate	547-63-7	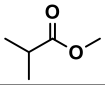	102.1	Bacteria
26	α-Humulene	6753-98-6	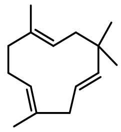	204.4	Plants
27	3-Methylthio-1-propanol	505-10-2	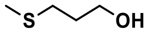	106.2	Bacteria
28	Anisole	100-66-3	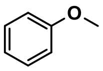	108.1	Plants
29	Benzyl alcohol	100-51-6	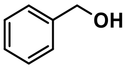	108.1	Plants
30	2-Acetyl-1*H*-pyrrole	1072-83-9	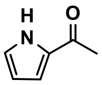	109.1	Plants
31	2-Ethylthiophene	872-55-9	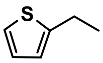	112.2	Bacteria
32	1,8-Cineole	470-82-6	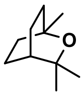	154.3	Plants
33	2-Heptanone	110-43-0	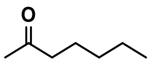	114.2	Plants
34	(*R*)-Sabinene	2009-00-9	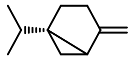	136.2	Plants

35	Benzyl cyanide	140-29-4	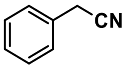	117.2	Plants
36	2-Phenethanamine	64-04-0	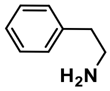	121.2	Bacteria
37	2-Phenylethanol	60-12-8	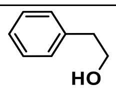	122.2	Bacteria
38	p-Hydroxy benzaldehyde	123-08-0	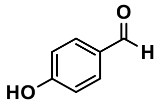	122.1	Bacteria
39	(2*E*,4*E*)-2,4-octadienal (OCT)	5577-44-6	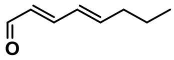	124.2	Insects
40	6-Methyl-5-hepten-2-one	110-93-0	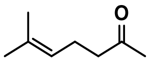	126.2	Insects
41	2-Acetylthiazole	24295-03-2	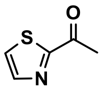	127.2	Bacteria
42	4-Octanone	589-63-9	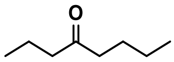	128.2	Plants
43	2-Octanone	111-13-7	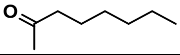	128.2	Plants
44	Ethyl isovalerate	108-64-5	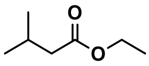	130.2	Plants
45	(1*S*)-3-Carene (CAR)	498-15-7	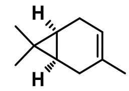	136.2	Plants
46	(*R*)-Limonene	5989-27-5	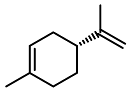	136.2	Plants
47	(*S*)-Limonene	5989-54-8	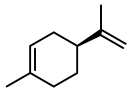	136.2	Plants
48	Camphene	79-92-5	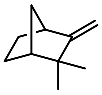	136.2	Plants
49	α-Pinene	7785-26-4	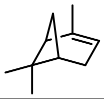	136.2	Plants
50	β-Pinene	18172-67-3	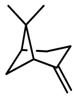	136.2	Plants

Table S-2: Repellence quantifications of tested compounds in *in vivo* assays by human hand landing numbers.

<table>
<tr><th>Compounds</th><th>MW</th><th>µL of 100% solution per cm²</th><th>nmole equivalent compound per cm²</th><th>Average landings (± st. dev)</th><th>DEET Pr(<[t])</th><th colspan="6">Single compound Pr(<[t])</th></tr>
<tr><td rowspan="3">Solvent control</td><td></td><td>0.2</td><td></td><td>52.8±6.9</td><td></td><td colspan="6"></td></tr>
<tr><td></td><td>0.04</td><td></td><td>53.3 ±13.7</td><td></td><td colspan="6"></td></tr>
<tr><td></td><td>0.01</td><td></td><td>54.7±9.3</td><td></td><td colspan="6"></td></tr>
<tr><td rowspan="3">N,N-Diethyl-3-methyl benzamide (DEET)</td><td>191.3</td><td>0.2</td><td>1042</td><td>0±0</td><td></td><td colspan="6"></td></tr>
<tr><td></td><td>0.04</td><td>208</td><td>0±0</td><td></td><td colspan="6"></td></tr>
<tr><td></td><td>0.01</td><td>52</td><td>1.8±1.4</td><td></td><td colspan="6"></td></tr>
<tr><td rowspan="3">Carvacrol (CRV)</td><td>150.2</td><td>0.2</td><td>1300</td><td>0.1±0.4</td><td>0.37737</td><td colspan="6"></td></tr>
<tr><td></td><td>0.04</td><td>260</td><td>0.5±1.1</td><td>0.23994</td><td colspan="6"></td></tr>
<tr><td></td><td>0.01</td><td>65</td><td>17.6±4.9</td><td>6.9E-16</td><td colspan="6"></td></tr>
<tr><td rowspan="3">Cumin alcohol (CA)</td><td>150.2</td><td>0.2</td><td>1300</td><td>0.1±0.4</td><td>0.04276</td><td colspan="6"></td></tr>
<tr><td></td><td>0.04</td><td>260</td><td>11.8±3.7</td><td>1.3E-06</td><td colspan="6"></td></tr>
<tr><td></td><td>0.01</td><td>65</td><td>20.1±6.5</td><td>1.3E-14</td><td colspan="6"></td></tr>
<tr><td rowspan="3">Isopropyl cinnamate (IPC)</td><td>190.2</td><td>0.2</td><td>1070</td><td>2.1±0.8</td><td>0.04835</td><td colspan="6"></td></tr>
<tr><td></td><td>0.04</td><td>214</td><td>-</td><td></td><td colspan="6"></td></tr>
<tr><td></td><td>0.01</td><td>54</td><td>-</td><td></td><td colspan="6"></td></tr>
<tr><td rowspan="3">Linalyl acetate (LA)</td><td>196.3</td><td>0.2</td><td>920</td><td>14.6±8.1</td><td>0.00091</td><td colspan="6"></td></tr>
<tr><td></td><td>0.04</td><td>184</td><td>-</td><td></td><td colspan="6"></td></tr>
<tr><td></td><td>0.01</td><td>46</td><td>-</td><td></td><td colspan="6"></td></tr>
<tr><td rowspan="3">(2E,4E)-2,4-Octadienal (OCT)</td><td>124.2</td><td>0.2</td><td>1410</td><td>0±0</td><td></td><td colspan="6"></td></tr>
<tr><td></td><td>0.04</td><td>280</td><td>1.1±1.5</td><td>0.06309</td><td colspan="6"></td></tr>
<tr><td></td><td>0.01</td><td>70</td><td>47.1±7.0</td><td>8.6E-25</td><td colspan="6"></td></tr>
<tr><td rowspan="3">(1S)-3-Carene (CAR)</td><td>136.2</td><td>0.2</td><td>1270</td><td>5.4±2.3</td><td>0.00181</td><td colspan="6"></td></tr>
<tr><td></td><td>0.04</td><td>254</td><td>-</td><td></td><td colspan="6"></td></tr>
<tr><td></td><td>0.01</td><td>64</td><td>-</td><td></td><td colspan="6"></td></tr>
<tr><td rowspan="3">CRV+CA</td><td rowspan="2"></td><td rowspan="2">0.02</td><td rowspan="2">130+130</td><td rowspan="2">0±0</td><td rowspan="2"></td><td colspan="3">CRV</td><td colspan="3">CA</td></tr>
<tr><td colspan="3">0.20708</td><td colspan="3">3.4E-07</td></tr>
<tr><td></td><td>0.005</td><td>32.5+32.5</td><td>3.8±3.7</td><td>0.02971</td><td colspan="3">1.8E-05</td><td colspan="3">3.7E-05</td></tr>
<tr><td rowspan="3">CRV+OCT</td><td rowspan="2"></td><td rowspan="2">0.02</td><td rowspan="2">130+140</td><td rowspan="2">0±0</td><td rowspan="2"></td><td colspan="3">CRV</td><td colspan="3">OCT</td></tr>
<tr><td colspan="3">0.20708</td><td colspan="3">0.04658</td></tr>
<tr><td></td><td>0.005</td><td>32.5+35</td><td>8.6±3.5</td><td>1.3E-09</td><td colspan="3">0.00087</td><td colspan="3">3.9E-09</td></tr>
<tr><td rowspan="3">CA+OCT</td><td rowspan="2"></td><td rowspan="2">0.02</td><td rowspan="2">130+140</td><td rowspan="2">0±0</td><td rowspan="2"></td><td colspan="3">CA</td><td colspan="3">OCT</td></tr>
<tr><td colspan="3">3.4E-07</td><td colspan="3">0.04658</td></tr>
<tr><td></td><td>0.005</td><td>32.5+35</td><td>12.9±3.8</td><td>8.0E-14</td><td colspan="3">0.02423</td><td colspan="3">8.3E-08</td></tr>
<tr><td rowspan="3">CRV+CA+OCT</td><td rowspan="2"></td><td rowspan="2">0.013</td><td rowspan="2">86.7+86.7 +93.3</td><td rowspan="2">0±0</td><td rowspan="2"></td><td colspan="2">CRV</td><td colspan="2">CA</td><td colspan="2">OCT</td></tr>
<tr><td colspan="2">0.20708</td><td colspan="2">3.4E-07</td><td colspan="2">0.04658</td></tr>
<tr><td></td><td>0.0033</td><td>21.7+21.7 +23.3</td><td>4.9±2.9</td><td>0.00019</td><td colspan="2">4.3E-05</td><td colspan="2">9.6E-05</td><td colspan="2">4.5E-09</td></tr>
</table>

Table S-2. Statistically significant differences between antagonists and DEET control as well as between mixtures and single compounds at equivalent doses are those with Pr(<[0.05]). -, not examined.

Figure S-1

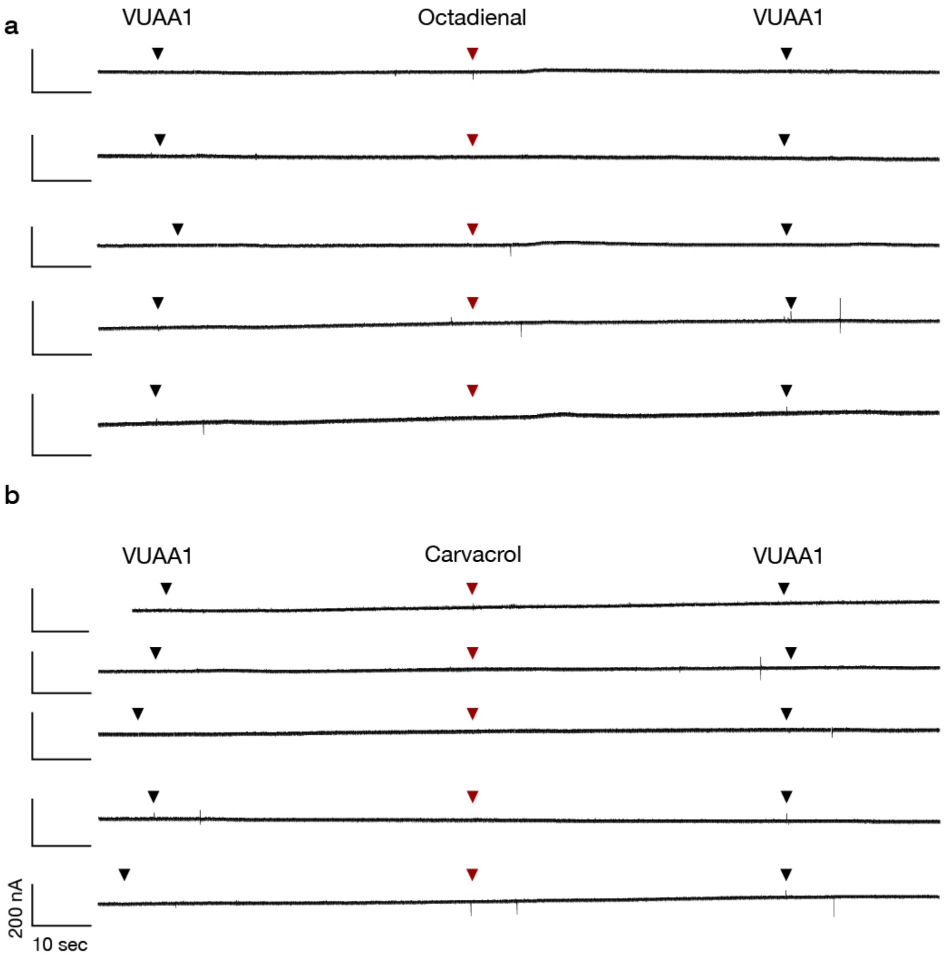

Figure S-1. VUAA1, octadienal and carvacrol do not elicit currents in water-injected oocyte controls. a) Water-injected oocytes do not display currents following exposure to $2x10^{-4}$ M octadienal (n = 5). **b**) Water-injected oocytes do not display currents following exposure to $2x10^{-4}$ M carvacrol (n = 5).

Table S-3: Antagonist IC_{50} values from binding competition assays shown in Figure 6. Antagonists IC_{50} values in the presence of 50µM OA compared to IC_{50} values in the presence of increasing OA concentrations (100 and 150µM). OCT exhibits a significant increase in IC_{50} concentration as OA presence is increased, while CRV and CA show no significant difference in their IC_{50} values as OA concentration is increased. Statistically significant differences are considered those with Pr(<[0.05]).

Compound	Concentration of OA (µM)	IC_{50} (µM)	OA 50µM Pr(<[*t*])
CRV	50	26.32 (pIC50: 4.58001±0.21732)	
	100	23.38 (pIC50: 4.63179±0.09524)	0.16712
	150	28.37 (pIC50: 4.54608±0)	0.55588
CA	50	84.67 (pIC50: 4.07226±0.23144)	
	100	83.17 (pIC50: 4.08243±0.28481)	0.71653
	150	77.79 (pIC50: 4.10898±0.17809)	0.23766
OCT	50	41.76 (pIC50: 4.37887±0.03444)	
	100	59.77 (pIC50: 4.22309±0)	0.04029
	150	116.76 (pIC50: 3.93263±0.26793)	2.73E-03

Table S-4: Agonist EC_{50} values from dose response curves shown in Figure 7. ORco agonist (ORcoRAM2) EC_{50} values in the absence and presence of 100µM of the three most potent antagonists, CRV, CA and OCT. In the presence of OCT, the OA EC_{50} exhibits a significant increase, while in the presence of CRV and CA no significant difference in EC_{50} values are observed. Statistically significant differences are those with Pr(<[0.05]).

**Compound**	**Antagonist Concentration (µM)**	**OA EC_{50} (µM)**	**No antagonist Pr(<[*t*])**
no antagonist		91.34<br>(pEC50: 4.0409±0.71927)	
CRV	100	97.2<br>(pEC50: 4.01241±0.56797)	0.63751
CA	100	96.1<br>(pEC50: 4.00222±0.94917)	0.64267
OCT	100	124.13<br>(pEC50: 3.90655±0.65211)	0.03093
